# Health-related quality of life: a retrospective study on local vs. microvascular reconstruction in patients with oral cancer

**DOI:** 10.1186/s12903-019-0760-2

**Published:** 2019-04-27

**Authors:** J. K. Meier, J. G. Schuderer, F. Zeman, Ch. Klingelhöffer, M. Hullmann, G. Spanier, T. E. Reichert, T. Ettl

**Affiliations:** 1 Department of Oral and Maxillofacial Surgery, University Medical Center Regensburg, 93042 Regensburg, Germany; 2 Center for Clinical Studies, University Medical Center Regensburg, Regensburg, Germany

**Keywords:** Oral carcinoma, Microvascular reconstruction, Health-related quality of life, University of Washington Quality of life questionnaire

## Abstract

**Background:**

New medicinal and surgical oncological treatment strategies not only improve overall survival rates but continually increase the importance of Health-Related Quality of Life (HRQOL). The purpose of this retrospective cross-sectional study was to analyze HRQOL of patients with oral squamous cell carcinoma after ablative surgery and to evaluate predictive factors for HRQOL outcome.

**Methods:**

The study included 88 patients with histologically confirmed oral squamous cell carcinoma of whom 42 had undergone local reconstruction (LR) and 46 microvascular reconstruction (MVR). During follow-up, all patients completed the University of Washington Quality of Life Questionnaire (UW-QOL) containing 12 targeted questions about the head and neck. Descriptive analyses were made for the tumor site, the T-stage, and adjuvant therapies. HRQOL was compared between the LR and the MVR group with parametric tests. Further analyses were impact of the tumor site, the T-status, and the time from surgery to survey on HRQOL. Statistics also included multivariate correlations and different interaction effects.

**Results:**

HRQOL in the LR group was ‘very good’ with 84.3 ± 13.7 and ‘good’ in the MVR group with 73.3 ± 16.5 points. The physical domains swallowing (*p* = 0.00), chewing (*p* = 0.00), speech (*p* = 0.01), taste (*p* = 0.01), and pain (*p* = 0.04) were significantly worse in the MVR group. An increase in the T-status had a significant negative effect on swallowing (*p* = 0.01), chewing (*p* = 0.01), speech (*p* = 0.03), recreation (*p* = 0.05), and shoulder (*p* = 0.01) in both groups. Regarding the tumor site and subsequent loss of HRQOL, patients with squamous cell carcinoma on the floor of the mouth had significantly worse results in the categories pain (*p* = 0.002), speech (*p* = 0.002), swallowing (*p* = 0.03), activity (*p* = 0.02), and recreation (*p* = 0.01) than patients with tumors in the buccal mucosa. Speech (*p* = 0.03) and pain (*p* = 0.01) had improved 1 year after surgery.

**Conclusion:**

Patients with flap reconstruction because of oral squamous cell carcinoma showed very good overall HRQOL. Outcomes for microvascular reconstruction were good, even in the case of larger defects. The T-status is a predictor for HRQOL. Swallowing, chewing, speaking, taste, and pain were the most important issues in our cohort.

Implementing HRQOL questionnaires for the assessment of quality of life could further increase the treatment quality of patients with oral cancer.

## Background

Disease-free overall survival seems to have been the most important measure of success of oncological treatment for decades. Over the past few years, however, functional and psychosocial rehabilitation has progressively become an essential secondary outcome [1–5]. In this regard, the model of Health-Related Quality of Life (HRQOL) was developed to assess patient function and well-being after oncological therapy and to gain structured insight into disease- and therapy-derived problems [2, 6, 7] HRQOL needs certainly to be seen as a complex multidimensional construct with a very individual character [1, 7, 8]. Different aspects and measurement instruments have been suggested to obtain comprehensive and comparable data on cancer patients [4–6]. For patients with head and neck cancer, targeted questionnaires on disease and site specificity have been developed [8]. Cancer originating from complex anatomical regions such as the oral cavity or the pharynx frequently requires extensive treatment with adverse effects on pivotal functions. Loss of swallowing, chewing, and speaking or having to deal with disfigured facial traits has an enormous physical and social impact on patients’ lives [8–11]. To avoid impairment and to restore functionality, microvascular and local flap reconstruction techniques have been developed that are implemented according to the surgeon’s expertise and the tumor characteristics [12–14]. In larger T-graded tumors, microvascular reconstruction, for instance with radial or fibula flaps, provides reliable functional and esthetic outcomes [9, 15]. Nevertheless, the therapeutic regimen is being constantly further developed, and data on oral cancer are still somewhat limited [8]. Further aspects of HRQOL have to be identified in the future to be able to use quality of life as a standardized outcome parameter after flap reconstruction and to enhance oncological outcome while minimizing postoperative handicaps [8, 10, 13].

The aim of this study was to measure HRQOL in patients with oral squamous cell carcinoma after oncological and reconstructive surgery. Predictors for HRQOL were identified by means of the targeted University of Washington Quality of Life Questionnaire (UW-QOL) followed by the evaluation of retrospective data.

## Methods

### Study design

The retrospective cross-sectional study was conducted at the Clinic of Oral and Maxillofacial Surgery of the University Medical Center Regensburg over a period of 18 months under the approval of the local Ethics Committee and according to the regulations of the protection of data privacy. Included patients were staged for squamous cell carcinoma of the oral cavity according to the current UICC TNM classification [16]. The nodal status was documented but not incorporated into this study. All patients had undergone oncological tumor resection with curative intent and immediate reconstruction after the staging process according to the current German guidelines for the treatment of cancer in the oral cavity [17]. Therefore, the common reconstructive techniques carried out were primary wound closure, different local flaps, and free tissue transfer. Microvascular reconstruction comprised radial forearm flaps, fibula flaps, anterior- lateral thigh flaps, lateral upper arm flaps, and iliac crest. Neck dissection and adjuvant therapies such as radiotherapy were initiated based on the decision of the local tumor board. ICD coding of the tumor sites was based on the documented anatomical subsites of the primary tumor localization.

All patients had completed the validated German Version of the University Of Washington Quality Of Life Questionnaire (UW-QOL) including 12 targeted head and neck categories at least 3 months after surgery [18–20]. Following Lowe et al., UW-QOL results were transformed into scores from 0 to 100; 0 to 20 was rated as ‘very bad’ HRQOL, 20 to 40 as ‘bad’, 40 to 60 as ‘moderate’, 60 to 80 as ‘good’, and 80 to 100 as ‘very good’ [20].

Inclusion criteria for the study were:

- Histologically confirmed oral squamous cell carcinoma,
- Oncological surgery with immediate local or microvascular reconstruction at our clinic over aperiod of 18 months, and
- Being disease-free at the time of the survey.

Exclusion criteria for the study were:

- Any other histological type of oral or extraoral carcinoma,
- Tumor site in the nasopharynx or hypopharynx,
- Unsuccessful local or free flap reconstruction,
- M1-status or planned palliative treatment, and
- Synchronous or metachronous second primary oral squamous cell carcinoma or tumor recurrence.

### Statistical analysis

Descriptive analyses were made for the tumor site, the T-stage, and adjuvant therapies. HRQOL was presented analogue to the questionnaire categories and score systems [18]. The following factors were analyzed with regard to their impact on HRQOL: the type of reconstruction (MVR or LR), the size of the tumor (T-status), the tumor site (floor of the mouth or buccal mucosa) and the postoperative survey time (3–6 months, 1 year, 1–2 years and ≥ 2 years). Univariate analyses were conducted using the chi squared and the Fisher’s exact tests and the Student’s *t* test. *P*-values < 0.05 were considered statistically significant.

ANOVAs were calculated to check multivariate correlations of reconstruction and other variables on HRQOL. To rule out interaction effects, the interaction term of reconstruction and other variables were included in the model. Subsequently, to rule out the possibility of another variable in the model changing the relationship between reconstruction and HRQOL, models were calculated for non-significant interaction terms, and main effects were investigated. Post hoc power analyses was conducted using G*Power 3.1.9.4.

All statistical analyses were conducted using IBM SPSS Statistics Version 21.0.

## Results

### Clinical characteristics of the patients

Overall, 88 patients had undergone oncological surgery with local or free microvascular reconstruction at our clinic over a period of 18 months. Regarding the T-classification, 52.2% of our patients had T1-status, 28.8% T2-status, and 18.1% T4-status. Only 1 patient had been treated for a T3-tumor. 80 patients (90.9%) underwent neck dissection, and 17 patients (19.3%) received adjuvant therapy. 46% of the patients had completed the survey within 1 year after surgery, 54% after 1 year and more. LR was mainly used for smaller T1-defects (69%), while MVR was almost equally used for T2-T4-defects. The clinical characteristics of our patients are summarized in Table 1. LR was possible in 42 patients, whereas 46 patients were received a microvascular transplant (MVR). Tumors on the tongue were mostly treated with local flap therapy (45.2%); in contrast, tumors on the floor of the mouth were primarily reconstructed with a microvascular transplant (56.5%). Primary tumor sites and the type of reconstruction are shown and encoded in Table 2.

**Table 1:** Clinical characteristics of the study participants

	Types of reconstruction		
*N* = 88	LR *n* = 42 (47.8%)	MVR *n* = 46 (52.2%)	Total *n* = 88
Sex			
Men	27 (64.2%)	31 (67.4%)	58 (65.9%)
Women	15 (35.7%)	15 (32.6%)	30 (34.1%)
Age	60.4 ± 12.0 y	58.9 ± 9.7 y	59.7 ± 10.8 y
T-status			
T1	29 (69.0%)	17 (33.0%)	46 (52.2%)
T2	9 (21.4%)	16 (34.8%)	25 (28.4%)
T3	1 (2.3%)	0	1 (1.1%)
T4	3 (7.1%)	13 (28.3%)	16 (18.1%)
Adjuvant therapy			
Radiotherapy	4 (9.5%)	5 (10.8%)	9 (10.2%)
Radio chemotherapy	1 (2.3%)	5 (10.8%)	6 (6.8%)
Chemotherapy alone	1 (2.3%)	1 (2.2%)	2 (2.3%)
No adjuvant therapy	36 (85.7%)	35 (76.1%)	71 (80.6%)
Time from surgery to survey			
3–6 months	12 (28.5%)	15 (32.6%)	26 (29.5%)
1 year	9 (21.4%)	5 (10.8%)	14 (15.9%)
1–2 years	5 (11.9%)	6 (13%)	11 (12.5%)
≥ 2 years	17 (40.4%)	20 (43.5%)	37 (42%)
Neck dissection			
Yes	37 (88.1%)	43 (93.5%)	80 (90.9%)
No	5 (11.9%)	3 (6.5%)	8 (9.1%)

*LR* local reconstruction, *MVR* microvascular reconstruction

**Table 2:** Primary tumor sites and applied reconstructive techniques

	Primary tumor site						
*n* = 88	Floor of mouth *n* = 34	Tongue *n* = 27	Buccal mucosa *n* = 13	Alveolar mucosa *n* = 10	Base of the tongue *n* = 3	Retromolar region *n* = 1	Total
ICD-Codes	C04.8	C02.0	C06.0	C03.9	C01	C06.2	
*LR n =* 42	*n =* 8	*n =* 19	*n =* 7	*n =* 6	*n =* 2	*n =* 0	
Primary wound closure	5	11	4	2	2	–	24
Local flap	1	8	2	4	–	–	15
Free skin	2	–	1	–	–	–	3
*MVR n =* 46	*n =* 26	*n =* 8	*n =* 6	*n =* 4	*n =* 1	*n =* 1	
Forearm flap	17	8	6	2	1	1	35
Fibula flap	5	–	–	2	–	–	7
Antero lateral thigh flap	1	–	–	–	–	–	1
Lateral upper arm flap	1	–	–	–	–	–	1
Iliac crest	2	–	–	–	–	–	2

*LR* local reconstruction, *MVR* microvascular reconstruction

### UW-QOL scores

**Fig. 1:**
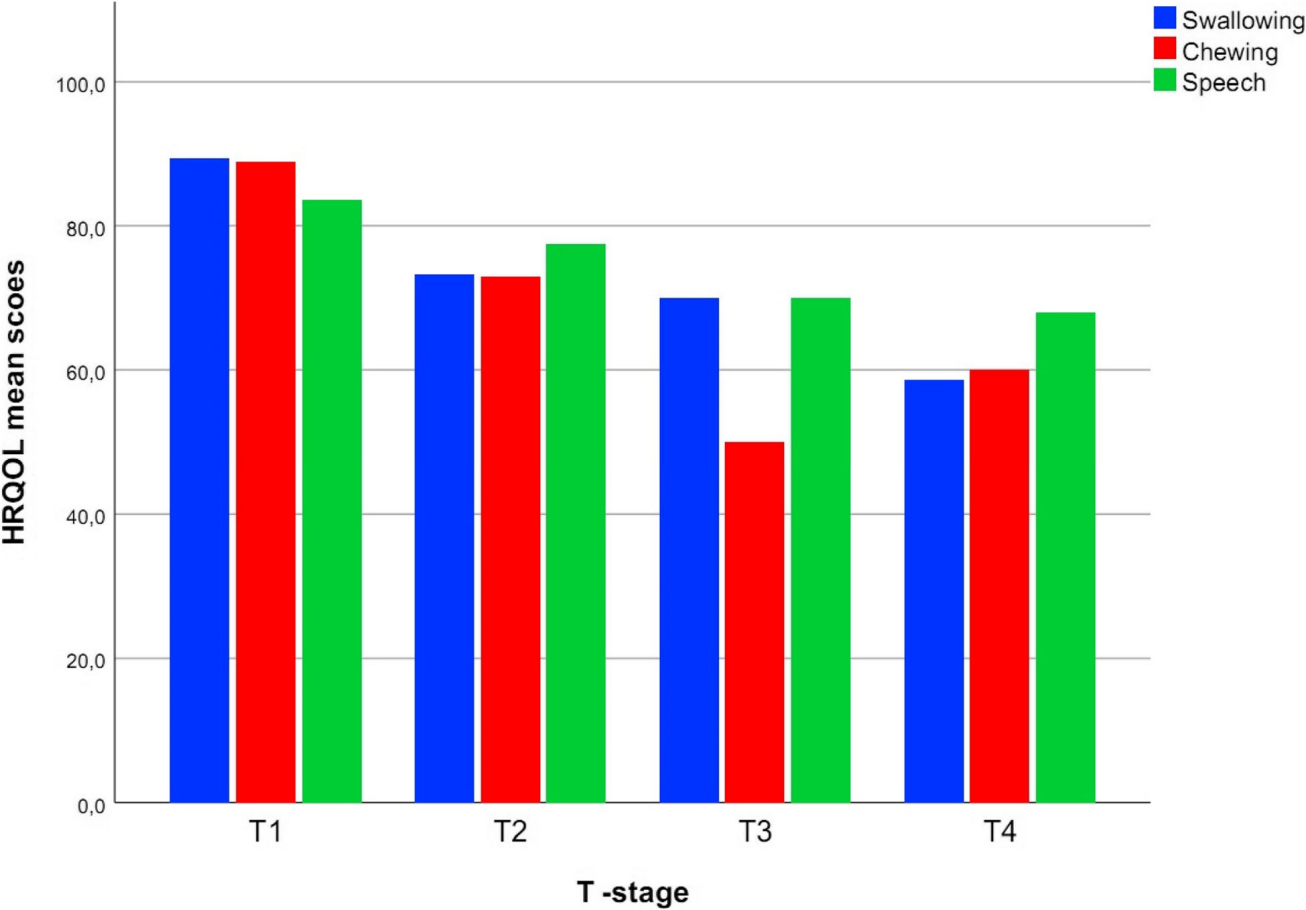
HRQOL mean scores plotted against the T-stage

The UW-QOL data shown in Table 3 refer to physical issues from appearance to taste and to social issues from pain to anxiety [20]. The comparison of the mean scores of the LR and MVR groups showed that the domains swallowing (*p* = 0.001), chewing (*p* = 0.000), speech (*p* = 0.011), taste (*p* = 0.014), and pain (*p* = 0.036) were significantly worse in the MVR group. The LR group showed ‘very good’ HRQOL with 84.3 ± 13.7, the MVR group only ‘good’ HRQOL with 73.3 ± 16.5 points (*p* = 0.001). Investigating the effects that tumor size might have on HRQOL, we found that increases in the T-stage significantly impaired swallowing (*p* = 0.01), chewing (*p* = 0.01), speech (*p* = 0.03), recreation (*p* = 0.05), and shoulder (*p* = 0.01) (Table 4 and Fig. 1). Investigations into the influence of the tumor site and subsequent loss of HRQOL showed that patients with squamous cell carcinoma on the floor of the mouth had significantly worse results in the categories pain (*p* = 0.002), speech (*p* = 0.002), swallowing (*p* = 0.03), activity (*p* = 0.02), and recreation (*p* = 0.01) than patients with tumors in the buccal mucosa. Regarding the time from surgery to survey, 42% of the patients completed the survey more than 2 years after surgery. Almost 30% of patients completed the survey 3 to 6 months after surgery. Pain was rated as significantly less 1 year (*p* = 0.01) and ≥ 2 years (*p* = 0.03) after surgery than after 3 to 6 months after surgery. Furthermore, the category speech (*p* = 0.01) was significantly improved 1 to 2 years and ≥ 2 years (*p* = 0.03) after surgery compared to the ratings after 1 year. Overall, the ratings of the HRQOL domains were not significantly improved less or equal 1 year from surgery to survey compared to ratings greater or equal 1 year from surgery to survey.

**Table 3:** Domains and mean scores of the University of Washington Quality of Life Questionnaire assessed globally and separately for the LR and MVR groups

UWQLQ		N	Score mean	SD	*P*-value
	GLOBAL	87	79.0	21.2	
Appearance	LR	41	82.3	20.3	0.172
	MVR	46	76.1	21.7	
	GLOBAL	87	78.9	26.0	
Swallowing	LR	42	88.6	22.9	0.001
	MVR	45	70	25.8	
	GLOBAL	86	78.4	26.0	
Chewing	LR	42	89.3	20.8	0.000
	MVR	44	68.2	26.6	
	GLOBAL	86	78.8	18.8	
Speech	LR	42	84.0	17.3	0.011
	MVR	44	73.9	19.1	
	GLOBAL	86	77.3	25.7	
Saliva	LR	42	80.7	24.9	0.235
	MVR	44	74.1	26.4	
	GLOBAL	87	76.0	27.6	
Taste	LR	42	83.6	20.8	0.014
	MVR	45	69.1	31.5	
	GLOBAL	87	81.3	24.8	
Pain	LR	41	87.2	21.0	0.036
	MVR	46	76.1	26.9	
	GLOBAL	88	76.1	23.0	
Activity	LR	42	80.9	21.9	0.060
	MVR	46	71.7	23.3	
	GLOBAL	87	77.8	26.2	
Recreation	LR	42	82.7	26.8	0.095
	MVR	45	73.3	25.2	
	GLOBAL	80	78.7	30.8	
Shoulder	LR	38	84.5	24.7	0.115
	MVR	42	73.6	34.9	
	GLOBAL	87	78.4	22.6	
Mood	LR	42	83.3	20.4	0.051
	MVR	45	73.9	23.8	
	GLOBAL	87	81.9	18.6	
Anxiety	LR	42	84.3	15.2	0.263
	MVR	45	79.8	21.4	

*LR* local reconstruction, *MVR* microvascular reconstruction

*P*-values = comparison of mean values LR/MVR for two independent samples using the t-test

**Table 4:** Effect of the increase in the T-stage on HRQOL domains

Appearance	*p* = 0.24
Swallowing	*p* = 0.01
Chewing	*p* = 0.01
Speech	p = 0.03
Saliva	*p* = 0.3
Taste	*p* = 0.12
Pain	*p* = 0.94
Activity	*p* = 0.24
Recreation	*p* = 0.05
Shoulder	*p* = 0.01
Mood	*p* = 0.3
Anxiety	*p* = 0.3

Multivariate analyses of reconstruction, third variables, and HRQOL showed that interaction effects were not statistically significant (*p* > 0.05).

If third variables were added into the model, relationships between reconstruction and HRQOL only changed with regard to chemotherapy (*p* = 0.004). The T-status was a predictor for HRQOL scores but lost significance under multivariate observation (*p* = 0.051). All multivariate and interaction effects are presented in Table 5.

**Table 5:** Multivariate analyses for interaction effects (ANOVAs) and main effect on HRQOL

	Model	Variable	*P*-value
Interaction effects	1	reconstruction*age	0.351
	2	reconstruction*sex	0.173
	3	reconstruction*chemotherapy	0.672
	4	reconstruction*tumor site	0.079
	6	reconstruction*T-status	0.746
	7	reconstruction*neck dissection	0.771
	8	reconstruction*radiation	0.984
	9	reconstruction*postoperative time	0.163
Main effects	10	reconstruction	0.001
		age	0.916
	11	reconstruction	0.001
		sex	0.187
	12	reconstruction	0.003
		chemotherapy	0.004
	13	reconstruction	0.009
		tumor site	0.462
	15	reconstruction	0.012
		T-status	0.051
	16	reconstruction	0.002
		neck dissection	0.156
	17	reconstruction	0.003
		radiation	0.854
	18	reconstruction	0.002
		postoperative time	0.622

Level of significance *p* < 0.05

Postoperative time = time from surgery to survey

Post hoc power analyses showed a power of 0.98 on HRQOL tests. The effect size of T-status on HRQOL was moderate (d = 0.7).

## Discussion

Ablative surgery for oral cancer, for instance resection of parts of the tongue, the floor of mouth, or the jaw bones, has a major influence on the quality of life of patients. Reconstruction of the surgical defect is mandatory to restore function and appearance [10, 21]. Small defects can be reconstructed with local techniques such as primary closure, local flaps such as tongue flaps or nasolabial flaps, or in some patients with free skin transplants. Larger defects often require more elaborate techniques. For many years, pedicled flaps such as pectoralis major flaps were preferred. Over the past 20 years, reconstruction with free micro vascularized transplants has become standard, improving oncological and functional outcomes [11, 14]. Quality of Life has become a constant marker of success in any oncological treatment. Microvascular and local flap techniques have been widely used to restore functionality in patients with head and neck cancer. In this study, we investigated what parameters influence HRQOL and how quality of life differs between patients with complex and local reconstructions. We used the University of Washington Quality of Life questionnaire because it is well established, short, and easy to understand.

In our study, HRQOL was significantly influenced by the type of reconstruction. Patients treated with a microvascular flap scored significantly worse in various oral domains with an overall difference of 10 points. There seems to be a variety of reasons for the better health-related outcome of local reconstructive techniques in comparison to microvascular free flap reconstructions in our cohort. First of all, microvascular reconstruction may result in higher donor side morbidity and requires more complex postoperative management. In this study, radial forearm flaps and fibula flaps were the mostly used types of microvascular flaps. De Witt et al. could show in their study on donor side morbidity in radial forearm flaps that basically one out of three patients had substantial functional limb complaints 6 months after surgery [22]. Literature reports have described donor and recipient site complications in up to 45% of patients [23]. Furthermore, free fibula harvest is associated with multiple postoperative complications such as ischemia of the ipsilateral foot, wound dehiscence, and chronic pain [24–26] with a possible negative impact on global QOL scores. With regard to delicate intraoral function, the patients with microvascular reconstruction in our study had significantly impaired HRQOL in chewing (*p* = 0.00), swallowing (*p* = 0.001), speech (*p* = 0.011), and taste (*p* = 0.014). The consecutive destruction of nerve, bone, and muscle structures negatively influences chewing and speaking; additionally, intraoral flaps can often be relatively voluminous and inflexible, thus interfering with usual mastication and deglutition [27]. The T-status was also a predictor for HRQOL scores. Whereas 62% of MVRs were conducted in the case of T2-T4-sized tumors, 69% of local reconstructions were carried out in the case of T1 tumors. This trend may be due to the advanced T-status that naturally requires more extensive surgery to achieve R0 resection [28]. Nevertheless, we found that the predictive character of the T-status was decreasing under multivariate observation. Additionally, reconstructions of the floor of the mouth showed worse scores than those for buccal mucosa (Table 6). Particularly the floor of the mouth guarantees mobility of the tongue; thus, defects in this region significantly impair swallowing, mastication, and speaking. In contrast, reconstructed defects in the buccal mucosa are more associated with salivary retention and pain [21, 29]. The tongue is also essential for propulsion processes. Dwivedi et al. found low scores for swallowing in patients with cancer of the tongue [28, 30]. Furthermore, microvascular transplant techniques are time-consuming and require extended operating times, which could also affect outcomes, particularly in elderly patients [31, 32]. In addition, adjuvant therapies such as chemotherapy and neck dissection are more frequently used in the case of increasing UICC staging. In our study, the number of adjuvant therapies was higher in the MVR group, and chemotherapy had an impact on HRQOL. Among others, chemotherapy and radiotherapy lead to xerostomia and mucositis which could explain the increasingly impairment in swallowing, chewing, speech, and taste as well as the increased level of pain [33, 34]. In contrast, in adequate defects, local reconstructive approaches allow the use of local tissue anatomy, which is not only usually followed by uncomplicated healing but also avoids nutritional graft problems because no vessel anastomosis is required. The procedures are quick to carry out and may even be achieved under local anesthesia in selected patients, facilitating favorable outcomes [35].

**Table 6:** Difference in HRQOL between carcinoma on floor of the mouth and carcinoma in the buccal mucosa

Appearance	*p* = 0.6
Swallowing	*p* = 0.03
Chewing	*p* = 0.09
Speech	*p* = 0.00
Saliva	*p* = 0.6
Taste	*p* = 0.8
Pain	*p* = 0.00
Activity	*p* = 0.02
Recreation	*p* = 0.01
Shoulder	*p* = 0.2
Mood	*p* = 0.3
Anxiety	*p* = 0.9

Level of significance *p* < 0.05

Two key elements of studies observing HRQOL are evaluating postoperative time and the point of interrogation [36]. Assuming that oncological treatment itself is an extraordinary experience for patients in generally reduced health, we started HRQOL evaluation at least 3 months after surgery. Since data were only collected postoperatively, we had no baseline of physical and social functioning. Previous studies have shown that HRQOL experiences sometimes change over the first 12 months after surgery. However, such aggravations seem to stabilize within 1 year after surgery and can be used as a long-term indicator [5, 7, 10]. Comparing domains with regard to the point in time the survey was carried out, we found differences in the category pain that had improved within the first year and two years after surgery. The category speech had also improved 1 year after surgery. No interaction effects were found between postoperative time and HRQOL under reconstructive therapy. The literature lacks information on postoperative oral functioning. Naturally, postoperative healing processes and functional restitution take their time, so that the already mentioned period of 1 year seems to be realistic for the healing process.

Finally, our patients with microvascular reconstruction nevertheless showed global scores of 73.3, which is still considered good quality of life. Markkanen-Leppänen et al. found scores comparable to our findings [37]. In consideration of these findings, there are reasons to opt for a microvascular graft in the oral cavity. MVR allows greater flexibility in both planning and implementation. Septocutaneous ALT flaps are used because of their enormous flexibility and comparably low donor site morbidity, especially in comparison to pedicled pectoralis major flaps that are associated with greater aesthetic donor site issues, particularly in female patients with breast deformation [38–41]. Furthermore, ALT flaps enable wider tumor resections with R0 margins as well as sufficient defect closures [9, 10]. To improve outcome for oncological patients, it seems necessary to implement HRQOL assessment in preoperative planning and patient management.

This study has several limitations. First of all, because of the unavailability of pre-treatment data, UW-QOL scores could not be compared with preoperative functioning scores to evaluate if our results reflect long-term HRQOL. Since we only used one targeted head and neck questionnaire but oncological patients are treated interdisciplinary, our results could have been influenced by other medical conditions. Furthermore, HRQOL should be assessed in a larger cohort to reach better uniform distribution. In the future, it seems reasonable to include general cancer questionnaires in the assessment setup. This in mind, we accept that our results only allow limited conclusions.

## Conclusion

Patients’ reconstruction with microvascular flaps after oral squamous cell carcinoma has good postoperative HRQOL. The T-status is a predictor for HRQOL. Swallowing, chewing, speaking, taste, and pain were the most important issues in our cohort.

## Abbreviations

HRQOL: Health-Related Quality of Life
LR: Local reconstruction
MVR: Microvascular reconstruction
UWQLQ: University of Washington Quality of Life Questionnaire
